# Prostatic tuberculosis: case report

**DOI:** 10.1590/S1516-31802008000400007

**Published:** 2008-07-03

**Authors:** Daniel Sáenz-Abad, Santiago Letona-Carbajo, José Luis de Benito-Arévalo, Isabel Sanioaquín-Conde, Francisco José Ruiz-Ruiz

**Keywords:** Tuberculosis, urogenital, Prostate, Immunocompetence, Diagnosis, Antitubercular agents, Tuberculosis urogenital, Próstata, Inmunocompetencia, Diagnóstico, Agentes antituberculosos

## Abstract

**CONTEXT:**

Tuberculosis of the prostate has mainly been described in immunocompromised patients. However, it can exceptionally be found as an isolated lesion in immunocompetent patients.

**CASE REPORT:**

We report a case of prostatic tuberculosis in a young, healthy and immunocompetent patient with unremarkable findings from intravenous urographic examination. Computed tomography showed an abscess in the prostate and *Mycobacterium tuberculosis* was isolated in a urine culture. Treatment with isoniazid, rifampin and pyrazinamide was successful.

## RESUMEN

**CONTEXTO:**

La tuberculosis prostática ha sido principalmente descrita en pacientes inmunodeprimidos. Sin embargo, es excepcional como lesión aislada en pacientes inmunocompetentes.

**CASO CLINICO:**

Describimos el caso de un varón sano e inmunocompetente con tuberculosis prostática y hallazgos irrelevantes en la urografía de eliminación realizada. La tomografía computerizada (TC) mostró un absceso en próstata, aislándose en el urocultivo *Mycobacterium tuberculosis*. El tratamiento con isoniazida, rifampicina y pirazinamida fue exitoso.

## INTRODUCTION

Genitourinary tuberculosis is a common type of extrapulmonary tuberculosis. The kidneys, ureter, bladder or genital organs are usually involved. Tuberculosis of the prostate has mainly been described in immunocompromised patients.^1^ However, it can exceptionally be found as an isolated lesion in immunocompetent patients. We report a case of prostatic tuberculosis in a young, healthy and immunocompetent patient with unremarkable findings from intravenous urographic examination.

## CASE REPORT

**Figure 1:**
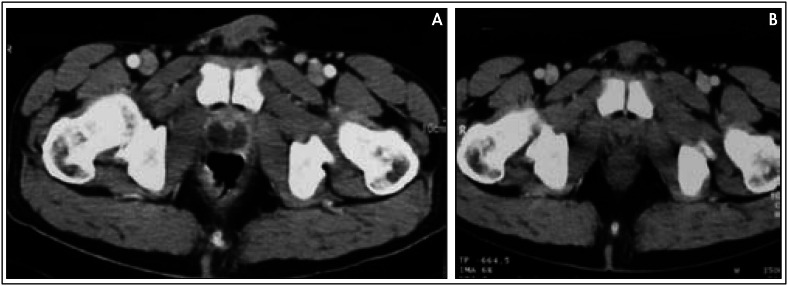
A. Computed tomography examination showing a large prostatic abscess with necrosis. B. Computed tomography examination after forty days of treatment, showing a normal size prostate gland with a minimal necrotic abscess.

A 36-year-old man who was born in Senegal was admitted to our hospital with a 12-month history of fever and fatigue and weight loss of 10 kg. He had no other medical problems and he reported not having traveled anywhere during the last two years. On examination, he was febrile. The physical examination otherwise revealed no abnormalities. His white blood cell count was 4,100/mm^3^, hematocrit 34% and erythrocyte sedimentation rate 80 mm/h. Urinalysis showed pyuria and no abnormalities were found on a chest radiograph. The blood and urine cultures were negatives and so was the HIV-antibody test. A tuberculin skin test was positive. Thoracoabdominal computed tomography (CT) examination revealed a large prostatic abscess with necrosis, while the other structures of the genitourinary system were normal (Figure 1a). Several days later, we were able to isolate *Mycobacterium tuberculosis* by urine culture in Lowenstein-Jensen medium. Finally, we performed an intravenous urographic examination, without finding any abnormalities in other structures of the urinary tract.

The patient was treated with isoniazid, rifampin and pyrazinamide for two months and he continued with isoniazid and rifampin for a further ten months. After this treatment, CT examination showed that the prostate had returned to its normal size and the necrotic abscess had decreased dramatically (Figure 1b).

## DISCUSSION

Changing patterns of population migration and the development of large pools of immunocompromised individuals has reversed the downward trend of tuberculosis.^2,3^ Today, extrapulmonary tuberculosis is becoming increasingly common, especially involving the lymphatic system, pleura and urogenital tract.^3^ Extrapulmonary sites are involved in 50% to 70% of immunocompromised patients, especially HIV patients. Genitourinary tuberculosis accounts for 5-10% of extrapulmonary cases in developed countries and 15-20% in developing countries. Nevertheless, isolated tuberculous prostatic abscesses are uncommon, especially in immunocompetent patients. *M. tuberculosis* is the most common pathogen involved, but others such as *M. kansasii* or *fortuitum* have been described. It is thought that tuberculous involvement of the prostate is usually the result of hematogenous spreading, although this can also occur as a result of descent of the organism from the kidneys or local spreading from the genital tract. Although sexual transmission of *M. tuberculosis* has been reported, it is extremely rare.^1^

Serial urine and semen cultures have a sensitivity of 50%. The polymerase chain reaction (PCR) test has been extensively used because it is a sensitive, specific and rapid technique. Although sterile pyuria is a classic feature of genitourinary tuberculosis, positive cultures for pyogenic organisms may lead to misdiagnosis. Pyuria plus hematuria with sterile cultures is a common urinary finding and intravenous pyelography examinations are abnormal in most cases of genitourinary tuberculosis.^4,5^ In some patients, prostate-specific antigen (PSA) may be elevated. Imaging studies help to locate and determinate the presence of concurrent tuberculosis in other organs. Therefore, transrectal ultrasound, intravenous urography and chest X-ray should be considered. Ultrasound reveals enlargement of the gland with solitary or multiple hypoechoic zones of varying sizes inside it. Irregularity of the outline of these hypoechoic areas may also be noted.

CT scans or magnetic resonance imaging may be useful for differential diagnosis, and some characteristic findings from prostate tuberculosis cases have been published. CT provides direct viewing of intraprostatic lesions and reveals them as low-density areas with irregular borders. Contrast-enhanced CT demonstrates these lesions more clearly. Magnetic resonance imaging (MRI) may reveal low signal-intensity lesions suggestive of abscesses. Intravenous urographic examination is recommended because, in a high percentage of cases, renal tuberculosis is found in association.^2,3^ In our case, CT scans showed an abscess with central necrosis that improved after treatment. These characteristics may be considered diagnostic for tuberculosis. Nonetheless, the definitive diagnosis was given by microbiological findings. Faced with findings of genitourinary tuberculosis, physicians should ensure that pulmonary involvement can be ruled out.

In conclusion, in this patient, the symptoms suggested the presence of tuberculosis. However, it is exceptional for the prostate alone to be affected, as an isolated lesion in the genitourinary tract of an immunocompetent patient.
